# Size dependent nanomechanics of coil spring shaped polymer nanowires

**DOI:** 10.1038/srep17152

**Published:** 2015-11-27

**Authors:** Shota Ushiba, Kyoko Masui, Natsuo Taguchi, Tomoki Hamano, Satoshi Kawata, Satoru Shoji

**Affiliations:** 1 Department of Applied Physics, Osaka University, 2-1 Yamadaoka, Suita, Osaka 565-0871, Japan; 2 Department of Engineering Science, The University of Electro-Communications, 1-5-1 Chofugaoka, Chofu, Tokyo 182-8585 Japan

## Abstract

Direct laser writing (DLW) via two-photon polymerization (TPP) has been established as a powerful technique for fabrication and integration of nanoscale components, as it enables the production of three dimensional (3D) micro/nano objects. This technique has indeed led to numerous applications, including micro- and nanoelectromechanical systems (MEMS/NEMS), metamaterials, mechanical metamaterials, and photonic crystals. However, as the feature sizes decrease, an urgent demand has emerged to uncover the mechanics of nanosized polymer materials. Here, we fabricate coil spring shaped polymer nanowires using DLW via two-photon polymerization. We find that even the nanocoil springs follow a linear-response against applied forces, following Hooke’s law, as revealed by compression tests using an atomic force microscope. Further, the elasticity of the polymer material is found to become significantly greater as the wire radius is decreased from 550 to 350 nm. Polarized Raman spectroscopy measurements show that polymer chains are aligned in nanowires along the axis, which may be responsible for the size dependence. Our findings provide insight into the nanomechanics of polymer materials fabricated by DLW, which leads to further applications based on nanosized polymer materials.

Since direct laser writing (DLW) via two-photon polymerization (TPP) was invented in 1997^1^, this technique has been established as a powerful tool for fabrication and integration of nanoscale components, as it enables the production of three dimensional (3D) polymer objects with feature sizes far beyond the diffraction limit of light^2^,^3^,^4^, which thereby expands the possible applications of polymer micro/nano structures, including micro- and nano-electromechanical systems (MEMS/NEMS)^5^,^6^, electromagnetic and mechanical metamaterials^7^,^8^, 3D photonic devices^9^, photonic crystals^10^, and soft or flexible micro/nano optics^11^. In addition, this technique has been applied with various materials, such as commonly-used photoresists like SCR-500^2^ and SU-8^12^, poly(methyl methacrylate) (PMMA)^13^, biopolymer^9^,^11^,^14^, hydrogels^15^, as well as nano-composites including carbon nanotubes^16^,^17^, semiconductor nanoparticles^18^, metallic nanoparticles^19^, and magnetic nanoparticles^20^. In contrast to the rapid progress on fabrication technology and its applications, however, fundamental characteristics such as mechanical properties of nanosized polymer materials still remain challenges.

Here, we present the mechanics of cross-linked PMMA nanowires formed into coil springs fabricated by the DLW technique. PMMA has been one of the most widely used polymers in industrial and academic fields. In addition, there are some studies about the mechanical properties of PMMA of bulk size^21^ and that of submicron size^22^. It is found in our study that even the nanocoil springs exhibit a linear-response against applied forces, following Hooke’s law, as revealed by compression tests using an atomic force microscope (AFM). Further, the elasticity of the polymer material is found to become significantly greater as the wire radius is decreased from 550 to 350 nm. Polarized Raman spectroscopy measurements suggest that polymer chain alignment in nanowires may be attributed to the size dependence.

## Results

### Fabrication of coil spring shaped polymer nanowires

**Figure 1:**
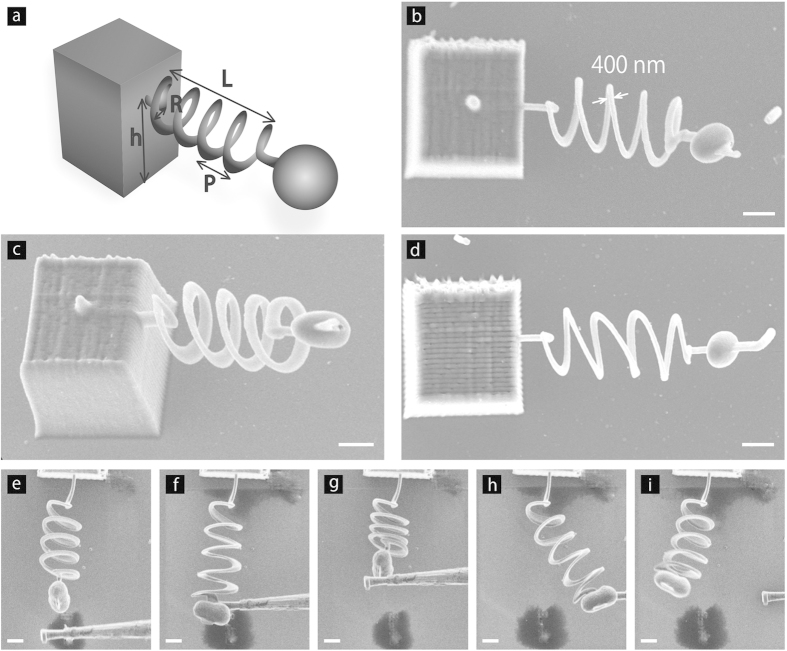
Polymer nanocoil springs fabricated by DLW through TPP lithography. (**a**) Schematic showing the dimensions of a polymer nanocoil spring. The coil radius, *R*, pitch, *P*, length of the spring, *L*, and the number of turns, *N*, are 2.5 μm, 2.0 μm, 13 μm, and 4, respectively. (**b**,**c**) SEM images of a polymer nanocoil spring. (**b**,**c**) are top and perspective views, respectively. (**d**) SEM image of a nanocoil spring supported by an additional polymer pole. (**e–i**) FIB images of the spring before loaded (**e**), stretched (**f**), compressed (**g**), bent (**h**), and recovered (**i**). All scale bars are 2 μm.

We use a DLW technique to fabricate nanocoil springs made of cross-linked PMMA (see Methods for more details). Figure 1a shows the dimensions of the spring. The coil radius, *R*, pitch, *P*, length of the spring, *L*, and the number of turns, *N*, are 2.5 μm, 2.0 μm, 13 μm, and 4, respectively. One end of the spring is attached to a bead of 1.0 μm radius. Figure 1b,c display one of the springs, with a wire width of 400 nm. An additional support was introduced, as shown in Fig. 1d, to prevent the springs from collapsing, when the wire width was narrower than 420 nm (Supplementary Fig. S-2). Owing to the additional support, springs with a lateral wire width as low as 188 nm at the thinnest point were fabricated (Fig. S-2). After fabrication, the supports were ablated using either a femtosecond pulsed near-infrared (NIR) laser beam or a focused ion beam (FIB). The springs were free-standing even after the removal of the supports, as shown in Fig. 1a–c. They exhibited properties of stretch, compress, bend, and recover, against forces applied through a manipulator, clearly indicating that the spring behaves as a conventional spring (Fig. 1e–i and Movie S1).

### Compression tests of coil springs

**Figure 2:**
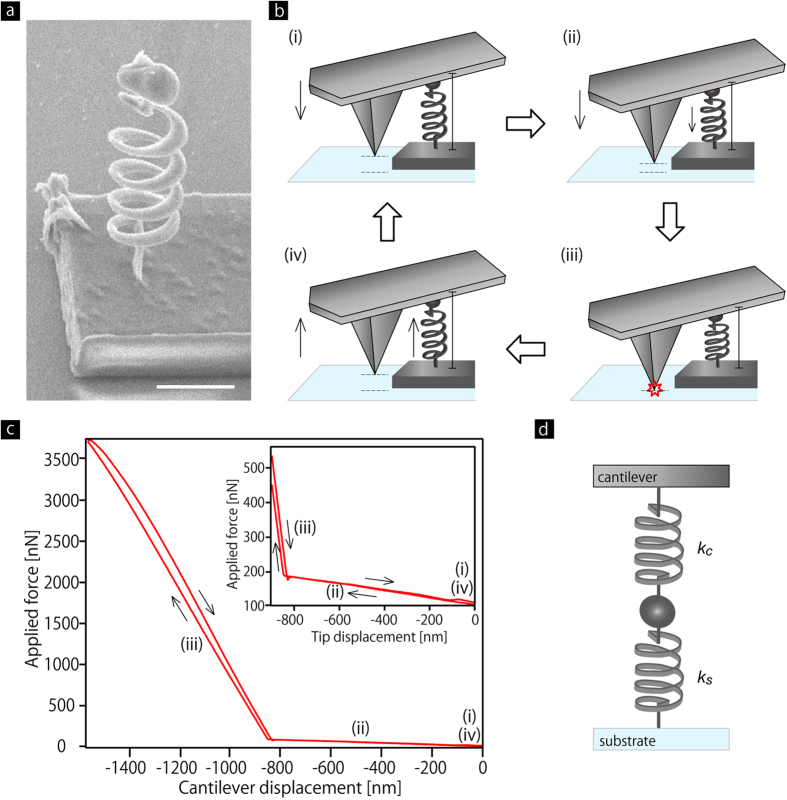
Compression tests on polymer nanocoil springs using an AFM. (**a**) SEM image of a vertically free-standing polymer nanocoil spring. The scale bar is 5 μm. (**b**) Procedure showing a compression test using AFM. (**c**) A representative force curve taken from one of the springs showing the applied force on the cantilever with respect to the position. The annotations in the graph correspond to the illustrations in (**b**). Inset to (**c**) The enlarged graph in the region of 0 to −800 nm showing a moderate linear slope. (**d**) Illustration showing the system regarded as a series of two springs. *k*_s_ and *k*_c_ represent the spring constant of the polymer spring and that of the AFM cantilever (13 N/m), respectively.

The nanomechanics of the springs were quantitatively characterized through compression tests. We fabricated vertically standing springs with the sizes as described above (see Methods for more details). Figure 2a shows one of the springs that stably and vertically stands without any distortion and bend. Compression tests were carried out on this spring using an atomic force microscope (AFM), as illustrated in Fig. 2b (see also Methods). It should be noted here that we deliberately press the spring with the flat surface of the cantilever, not with the tip apex, aiming to exert a force uniaxially to the spring and to avoid deformation of the bead. The cantilever moved downward (upward) for 1600 nm, corresponding to a monotonic load (unload) in compression. Figure 2c shows a representative force curve that records the applied force on the cantilever with respect to the cantilever position. The force curve shows two slopes, which are divided at a position of *z* = −840 nm where the tip apex contacts to the glass substrate: (i) moderate slope in the range of *z* = 0 (initial position) to −840 nm, in which the cantilever contacts the spring alone (Fig. 2c, inset); and (ii) steep slope in the range of *z* = −840 to −1600 nm, in which the cantilever contacts both the spring and the glass substrate. The force curve shows no hysteresis on the moderate slope, indicating no plastic deformation of the spring during the test. The moderate slope also gives a clear indication that even the nanocoil spring follows a linear-response against the applied force, namely, the nanocoil spring abides by Hooke’s law. Therefore, in this moderate slope region, the system can be regarded as a series of two springs (Fig. 2d), and the spring constant of the coil spring, *k*_s_, is given by Equation (1);


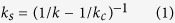


where *k* is an equivalent spring constant of the two springs. *k* is calculated by fitting the moderate slope with a linear function, to be 0.10 N/m. *k*_c_ is a spring constant of the AFM cantilever, which is 13 N/m after calibration using the effective length of the cantilever. By repeating compression tests on the same spring for more than 10 times, the average spring constant with the standard deviation is obtained, for example 0.10 ± 0.01 N/m. There is no plastic deformation of the spring, and thus the compression tests are reproducible.

### Size dependent nanomechanics of polymer nanowires

**Figure 3:**
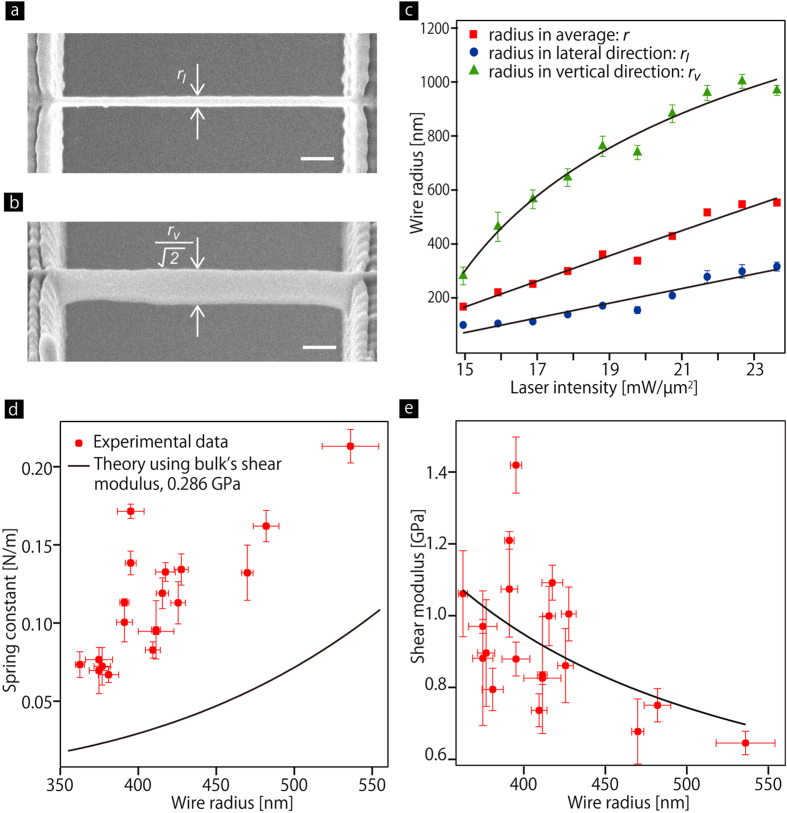
Size dependent nanomechanics of polymer nanocoil springs. (**a**,**b**) SEM images of a nanowire from top (**a**) and 45° views (**b**). Wire radii in lateral, *r*_l_, and in vertical, *r*_v_, are measured through SEM observation. Scale bars are 1 μm. (**c**) Wire radius, *r* (red), *r*_l_ (blue), and *r*_v_ (green) as a function of laser intensity. (**d**) Spring constant, *k*_s_, as a function of *r*, measured (symbols) and theory using bulk shear modulus, 0.29 GPa (solid line). (**e**) *G* of polymer nanowires in coil springs as a function of *r*.

To provide further insight on the nanomechanics of the polymer material, we investigated *k*_s_ with different wire radius, *r*. *r* is defined as *r* = (*r*_l *_ *r*_v_)^1/2^, where *r*_l_ and *r*_v_ are lateral and vertical radii, which were measured from a set of straight nanowires through scanning electron microscopy (SEM) observations (Fig. 3a,b). By fabricating nanowires with different laser intensity, *r* as a function of the laser intensity was obtained (Fig. 3c). The error bars represent non-uniformity of wire width, and the solid lines represent fitting curves^23^. We fabricated 18 coil springs with different *r* in the range of 350 to 550 nm, and performed the compression tests on those springs. Figure 3d presents *k*_s_ as a function of *r*, showing that *k*_s_ increases with decreasing *r*. Here, the spring constant is theoretically described by Equation (2)^21^:


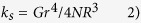


where *G* is the shear modulus of the material that is defined as the ratio of shear stress to shear strain, and the coil radius *R* = 2.5 μm, the number of turns *N* = 4, respectively. Using Equation (2) with the value of *G* of bulk sized cross-linked PMMA (0.29 GPa, see Methods), the theoretical spring constant is also provided in Fig. 3d. The error bars in vertical axis represent standard deviation of n = 10 measurements for each spring. There is a large gap between the experimental results and the theoretical curve, which derives from the size effect on *G*. To calculate *G* of the polymer nanowires, we substituted the size-dependent spring constant to the Equation (2) with the parameters *R* = 2.5 μm and *N* = 4. Figure 3e presents *G* as a function of *r* and an exponential fitting curve. This plot shows the significant increase with decreasing the radius smaller, although the error bars are large. It should be also noted here that relatively wider nanowires around 500 nm still exhibit a greater value of *G*, compared to bulk systems.

## Discussion

The size-dependent elasticity of the polymer nanowires reported here is opposed to our previous study^21^, where mechanical tests were performed in ethanol. As the size of polymer was a few hundreds nm, ethanol could penetrate into the entire polymer structure, leading to the reduction of the elasticity. The swelling becomes more significant with decreasing the size, so that the elasticity became smaller when the size became smaller. However, in the present study, the measurement was performed in air, and so the mechanism of the size dependent elasticity should be different.

The same trend of the size dependence has been found in many polymer nanowires, for example, polystyrene^24^, nylon 6.6 nanofibers^25^, polyacrylonitrile nanofibers^26^, polypyrrole nanotubes^27^, poly(2-acrylamido-2-methyl-1-propanesulfonic acid) nanofibers^28^, and polyvinyl alcohol fibers^29^. There are a lot of possible mechanisms of the size dependence such as surface chain orientations^24^, core/densely-packed-shell formation^29^, and supramolecular structure formation^25^.

**Figure 4:**
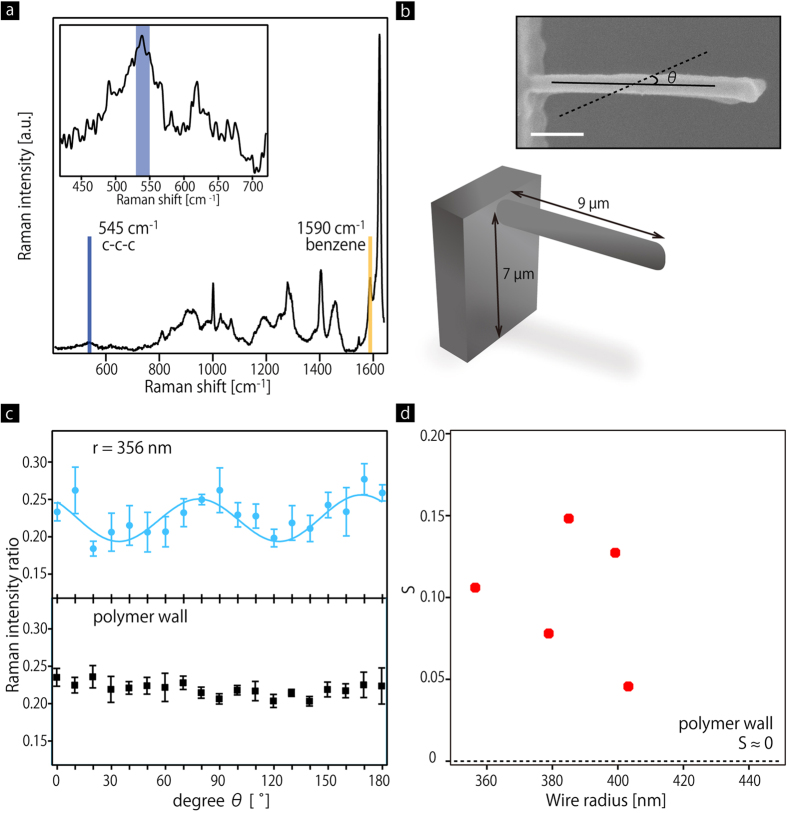
Polarized Raman microspectroscopy on polymer nanowires. (**a**) Raman spectrum of a nanowire, whose wire radius is 356 nm. Inset to (**a**) A magnified Raman spectrum around the peak at 545 cm^−1^. (**b**) Illustration of a cantilevered nanowire on the polymer wall. Inset to (**b**) Top view of a cantilevered nanowire. *θ* is defined as an angle between the polarization of the Raman excitation laser beam and the wire axis. Scale bar is 1 μm. (**c**) Raman peak intensity ratio of *I*_540_ to *I*_1590_ as a function of the angle *θ* taken from a wire, whose radius is 356 nm, and polymer wall (bulk) Solid line represents a fitting curve using Equation (3). (**d**) *S*, which represents strength of the alignment, as a function of wire radius. The dotted line represents *S* for bulk systems.

As a possible factor for the size-dependence, we investigated alignment of polymer chain networks in cantilevered nanowires with different wire radii, *r*, using polarized Raman microspectroscopy (see Methods). Polarized Raman spectra were measured with a different polarization angle, *θ*, between the polarization of the Raman excitation laser beam and the wire axis. Figure 4a displays a Raman spectrum taken from a cantilevered nanowire, whose wire radius, *r*_l_, is 356 nm (Fig. 4b), with a polarization angle *θ* = 0°. The spectrum contains two Raman peaks at 545 and 1590 cm^−1^: the former relatively broad peak is assigned to the C-C-C skeletal mode in PMMA and cross-linker, which constitutes the main chains of the structure; and the latter peak is assigned to benzene rings in the photo-initiator and photo-sensitizer, which are regarded as a polarization independent peak (Fig. S-3 and ref. 30,31). Therefore, *I*(*θ*), which expresses the peak intensity ratio of *I*_545_ to *I*_1590_ as a function of *θ*, represents the strength of the alignment of the main chains. *I*(*θ*) taken from 356 nm wide wire is plotted in Fig. 4c. *I*(*θ*) taken from the polymer wall sustaining the wires, *i.e.* the bulk, is also shown for comparison. The plots are fitted with a following fitting function (3)^32^


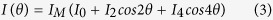


where *I*_M_, *I*_0_, *I*_2_, and *I*_4_ are the fitting parameters, respectively. The result clearly shows that *I*(*θ*) for the wire becomes greatest at *θ* = 0 and 180°, while it becomes smallest at *θ* = 45 and 135°, while *I*(*θ*) for the polymer wall is almost constant. This dependence indicates alignment of polymer chain networks. The strength of the alignment is quantitatively evaluated by introducing a parameter, *S*, which is defined by the following Equation (4)


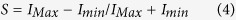


where *I*_Max_ and *I*_min_ are maximum and minimum values in *I*(*θ*), respectively. *S* becomes equal to 0 for random orientations, while it becomes getting larger as polymer chains are more strongly aligned. Using the Equation (4), *S* for the wires, whose radius is 356, 379, 385, 399, and 403 nm, respectively, are obtained, as shown in Fig. 4d. *S* for the bulk is assumed to be 0, as there is no specific angular dependence. This result indicates that polymer chain networks are aligned in the wires along the axis, and the alignment may be attributed to the enhancement in the mechanical properties. In addition, the result also shows that the alignment occurred even in the relatively wider wires, while the networks are randomly oriented in the bulk. This is a possible reason why relatively wider nanowires around 500 nm still exhibit a greater modulus, compared to bulk systems.

We presume that the alignment of polymer chain networks was induced by the DLW. There is a literature demonstrating the alignment of single-wall carbon nanotubes (SWCNTs) by using the DLW^17^. The DWL was performed on SWCNT-dispersed photo-resin, and it was found that SWCNTs were aligned in polymer nanowires along the axis. It was also found that the nematic order parameter, which represents the strength of the alignment of SWCNTs, increased from 0.2 to 0.4 with decreasing the wire width from 1000 nm to 400 nm. The possible reasons for the alignment of SWCNTs were (i) the spatial confinement, (ii) the volume-shrinkage during rinse and dry process after the DLW, leading to tensile force, and (iii) optical gradient force induced by the fabrication laser beam. The same alignment mechanisms, especially the spatial confinement and the volume-shrinkage, should also work in the present study, and thereby polarized Raman measurements showed alignment of the polymer chain networks along the nanowire axis. The alignment may be responsible for the enhancement in the shear modulus of the polymer nanocoil springs, as shown in our experimental result (Fig. 3e).

In summary, we have presented nanomechanics of free-standing polymer nanocoil springs fabricated by DLW. Compression tests using an AFM reveal that the springs exhibit a linear-response against applied forces in compression, following Hooke’s law in the same manner as conventional springs. The shear modulus is found to significantly increase from the bulk shear modulus with decreasing wire radius. Polarized Raman spectroscopy measurements show that polymer chains are aligned in nanowires, which may lead to the enhancement in the elasticity. In addition, we also demonstrated that the DLW enables one to characterize mechanical properties of free-standing, namely substrate-free, polymer nanostructures of which the size is precisely controlled. Thus, the DLW is useful not only for nanofabrication but also characterization of nanosized materials. Our work provides insight into the nanomechanics of polymer materials fabricated by DLW, which paves the way for further applications based on nanosized polymer materials.

## Methods

### Sample preparation

An UV-curable resin was prepared by mixing methyl methacrylate (MMA, Wako), cross-linker (DPE-6A, Kyoeisha Chemical Co., Ltd.), photo-initiator (Benzil, Wako), and photo-sensitizer (2-benzyl-2-(dimethylamino)-4′-morpholinobutyrophenon, Aldrich), at a ratio of 49, 49, 1.0, and 1.0 wt%, respectively (see Table S1 in Supplementary information). The resin exhibits strong absorption in the UV region, but no absorption in the visible or NIR regions (Fig. S-1).

### Fabrication

A Ti:sapphire femtosecond pulsed laser (Tsunami, Spectra-Physics, Newport Corp.) with a wavelength of 780 nm, a pulse width of 100 fs, and a repetition rate of 82 MHz, was used as the light source. The laser beam was focused, through an oil-immersion objective lens (100×, NA 1.4, Zeiss), onto the UV-curable resin casted on a glass substrate which was placed on a three dimensional piezoelectric stage. The stage moved the resin relative to the focus spot according to preprogrammed patterns. The typical laser intensity used in this study was 21 mW/μm^2^, and the exposure time was kept constant at 4 ms at each step. The wire widths were controlled by laser intensity. After the structures were fabricated, unsolidified resin was washed away using ethanol, and the ethanol was further replaced with supercritical CO_2_ fluid and then dried in Supercritical Rinser & Dryer (SCRD4, Rexxam Co., Ltd.), preventing the structures from collapsing during the dry process. Ablation of additional supports was carried out using either a NIR femtosecond pulsed laser or a FIB (FB2200, Hitachi High-Tech).

Vertically standing coil springs were obtained as follows. The springs, which are the same dimensions as Fig. 1a, were fabricated on a polymer wall of 50 μm length, 2.8 μm thickness, and 15 μm height. The wall was subsequently pushed down using a manipulator in FIB, and fixed onto the glass substrate by carbon deposition. The springs stably stood in vertical direction without any distortion and bend.

### Compression tests using an AFM

Compression tests on the vertically standing springs were carried out using an AFM (SPA-400, SII NanoTechnology Inc.) and an AFM cantilever (15 N/m, SI-DF20, SII NanoTechnology Inc.). The procedure of the compression test is shown in Fig. 2b: (i) the cantilever is initially set so that the cantilever was in contact with the bead of the spring. (ii) the cantilever compresses the spring but the cantilever tip does not contact the substrate, (iii) the tip contacts the glass substrate, and (iv) the cantilever returns to the initial position. The effective length of the cantilever, where the bead was in contact with the cantilever, was ~195 μm, while the total length of the cantilever was 225 μm, resulting in calibration of the spring constant to be 13 N/m. The cantilever moved downward for 1600 nm, exerting monotonically increasing compression on the spring, and then back to the initial position (Fig. 2b). A force curve was recorded during the cycle with a measurement duration of a few seconds (Fig. 2c).

### Tensile tests on bulk sized cross-linked PMMA

Cross-linked PMMA films were prepared from the same UV-curable resin described above under UV lamp illumination. The tensile tests, which were conducted in Japan Chemical Innovation and Inspection Institute, measured the Young’s modulus, *E*, and Poisson’s ratio, *ν*, as 813 MPa and 0.42, respectively. Therefore, the bulk shear modulus, *G*, is obtained as 0.29 GPa using Equation (5).


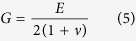


This value is used for the discussion.

### Polarized Raman microspectroscopy measurements and analysis

A set of straight cantilevered nanowires, whose wire radius is in the range of 356 to 403 nm, were fabricated on a polymer wall. To investigate alignment of polymer chain networks in the wires, polarized Raman microspectroscopy experiments were conducted using a Raman microscope (Raman-11, Nanophoton Corp.). The excitation laser beam, with a wavelength of 532 nm, was linearly-polarized by passing through a polarizer, and focused onto a sample through an objective lens (100×, NA 0.9, Nikon). The polarization direction was controlled with a half-wave plate placed between the objective lens and the polarizer. Raman scattering was collected by the same objective lens, and the polarization direction was rotated to be the same direction as that of the incident light after passing through the half-wave plate. The laser intensity and exposure time were optimized to take Raman spectra with a sufficient signal-to-noise ratio.

### SEM observations

SEM observations were made using a Field Emission-Scanning Electron Microscope (FE-SEM, JSM-6330F, JEOL). Before SEM observation, the sample was Osmium-coated with a thickness of a few nm using an Osmium-coater (HPC-30W, Vacuum Device Inc.).

## Additional Information

**How to cite this article**: Ushiba, S. *et al.* Size dependent nanomechanics of coil spring shaped polymer nanowires. *Sci. Rep.* **5**, 17152; doi: 10.1038/srep17152 (2015).

## Supplementary Material

Supplementary Information

Supplementary Movie
