# Supplementary material for: Size dependent nanomechanics of coil spring shaped polymer nanowires
Source: Sci Rep. 2015 Nov 27;5:17152. doi: 10.1038/srep17152 (PMC4661696; doi:10.1038/srep17152)
Supplement: Supplementary Information [file srep17152-s1.pdf]

Supplementary Information

Size dependent nanomechanics of coil spring shaped polymer nanowires

*Shota Ushiba, Kyoko Masui, Natsuo Taguchi, Tomoki Hamano, Satoshi Kawata and Satoru Shoji**

Preparation of an UV-curable resin.

An UV-curable monomer is prepared by mixing a monomer, methyl methacrylate (MMA, Wako), cross-linker (DPE-6A, Kyoeisha Chemical Co., Ltd.), photo-initiator (Benzil, Wako), and photo-sensitizer (2-benzyl-2-(dimethylamino)-4'-morpholinobutyrophenon, Aldrich), at a ratio of 49, 49, 1.0, and 1.0 wt%, respectively (Table S-1). Figure S-1 shows the absorption spectrum of the photo-resin. The photo-resin exhibits strong absorption in the UV region, but no absorption in the visible and near infrared (NIR) regions.

Table S1 | Chemical structure and recipe of materials.

	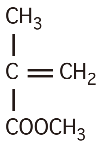 Monomer (MMA)	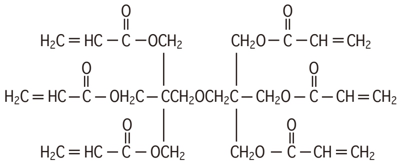 Cross-linker (DPE-6A)	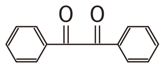 Photo-intiator (Benzil)	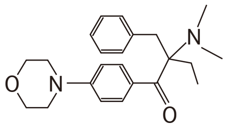 Photo-sensitizer
wt%	49	49	1	1

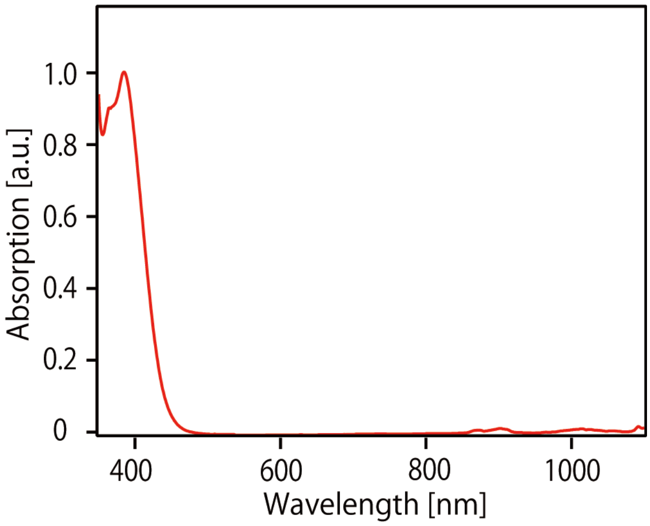

Figure S-1 | Absorption spectrum of the prepared UV-curable resin.

Fabrication of the thinnest polymer nanocoil springs.

We investigate how narrow the nanowires can be formed into a coil spring shape. Figure S-2 shows the lateral wire widths of the springs as a function of laser power. We found that the durability of the structure increases as the wire becomes wider, as

shown in Figure S-2, inset. Indeed, the springs remained without distortion when the width exceeds 420 nm. By introducing a support, springs with a lateral wire width as low as 188 nm at the thinnest point were fabricated. However, when further decreasing the laser power, the springs collapsed after the rinse and dry process despite the additional support, and eventually the springs were unable to be fabricated. This result clearly represents the durability of the structure.

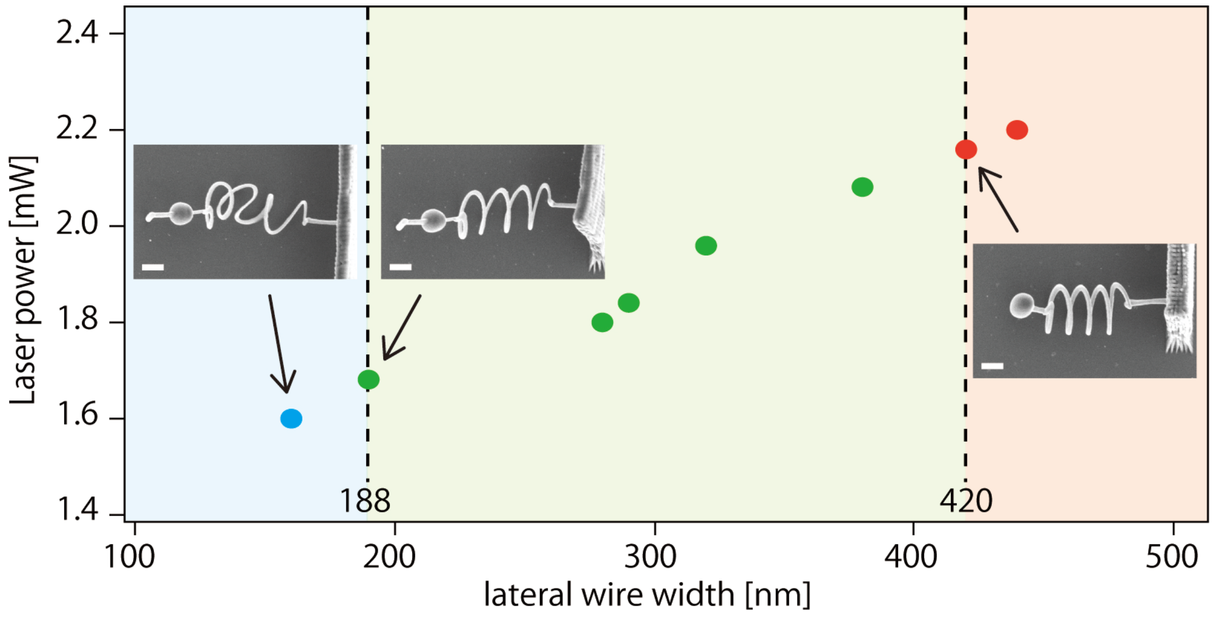

Figure S-2 | Polymer nanocoil springs fabricated with different laser powers. The graph represents the lateral wire widths of the springs as a function of laser power.

The springs shown as insets are fabricated with the corresponding laser power in the graph. Scale bars are 2 μm.

Raman spectroscopy of polymer materials.

Figure S-3 displays the Raman spectrum of each material: cross-linked PMMA fabricated by direct laser writing, PMMA (Sigma Aldrich), MMA, cross-linker, photo-initiator, and photo-sensitizer. According to this result and literatures[1,2], a Raman peak of cross-linked PMMA at 545 cm^{-1} is assigned to the C-C-C skeletal mode in PMMA and cross-linker, and a peak at 1595 cm^{-1} is assigned to a Raman mode of benzene rings in the photo-initiator and photo-sensitizer.

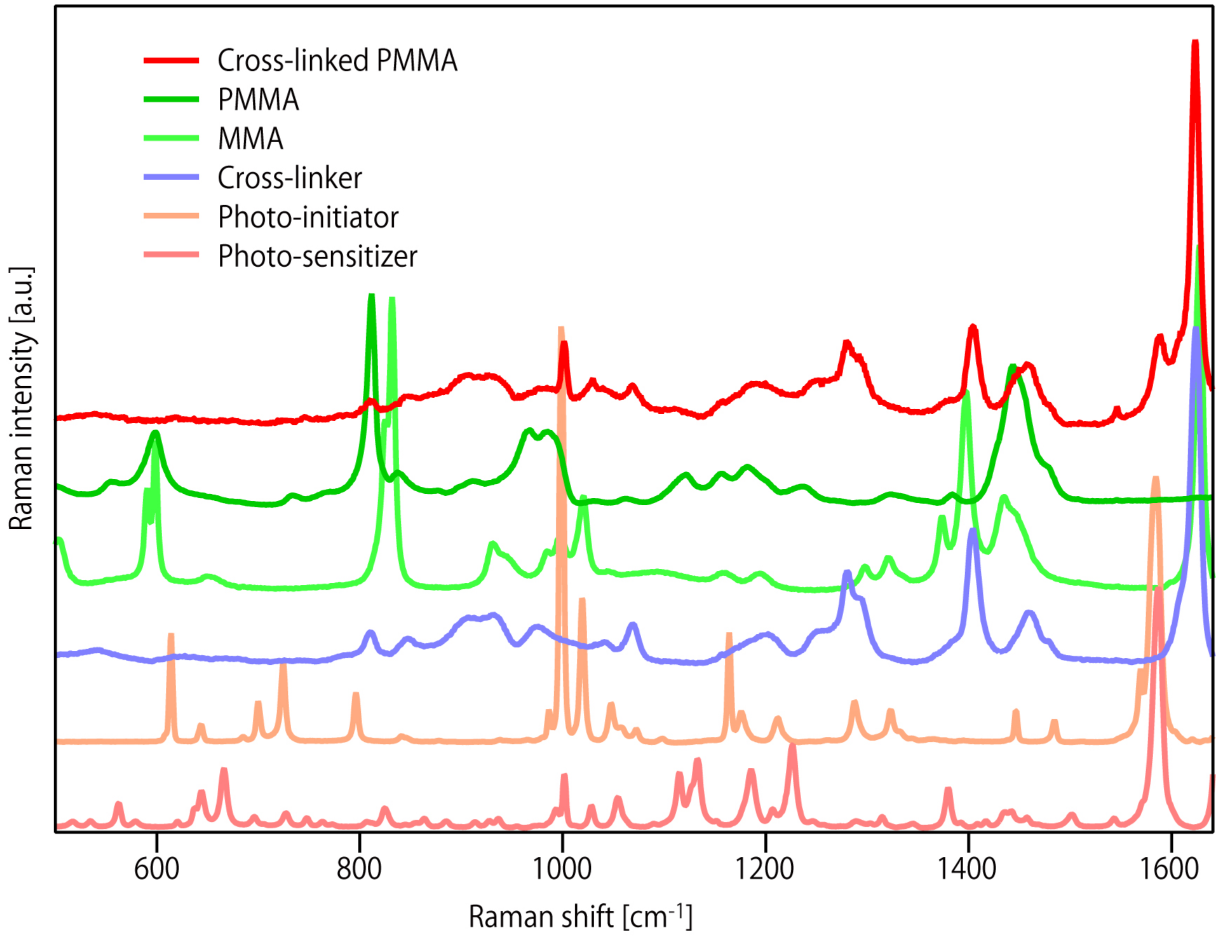

Figure S-3 | Raman spectrum of each material; cross-linked PMMA (red), PMMA (green), MMA (yellow-green), cross-linker (blue), photo-initiator (orange), and photo-sensitizer (pink). The Raman spectra were taken with an excitation wavelength of 532 nm.

References

1. Willis, H. A., Zichy, V. J. I. & Hendra, P. J. The laser-Raman and infra-red spectra of poly(methyl methacrylate). *Polymer* **10**, 737-746 (1969).

2. Socrates, G. *Infrared and Raman characteristic group frequencies: Tables and Charts.* (John Wiley & Sons. Ltd., UK, 2001).
